# The Coal Tar Products Again

**Published:** 1910-03

**Authors:** 


### The Coal Tar Products Again

(*National Druggist*, February, 1910.)

NOTE.—We publish this article in the interest of fairness. We do not indorse the statements it contains; indeed from some, perhaps many, of them we dissent in toto. But let all sides be heard.

IN a recent pamphlet, entitled "Antipyrine, Acetanilid and Phenacetin, Are they Harmful or Habit-Forming?" the author, Dr. Uriel S. Boone, an established physician of good standing in St. Louis, has furnished a valuable contribution to the long-drawn-out discussion of this important subject.

There are several points about this investigation of Dr. Boone's which distinguish it from almost every preceding canvass of the subject, all of which might be summed up in the statement that it bears every appearance of being a genuine search for the unvarnished truth, that it is conducted in a proper spirit of fair and open investigation, directed in the most reliable quarters, and that its results are presented in a fashion which makes his report peculiarly satisfying and convincing.

Dr. Boone has, as we think. rightly opined (sic) that "the hospitals and sanitariums of the United States would contain unbiased, unprejudiced evidence, unaffected by any thought of the result upon the drugs themselves;" and he has "selected them as the field of his investigation because they keep records of their cases which few physicians in private practice do, and because, if these drugs were habit-forming, many of their habitues would, naturally, go to hospitals and sanitaria for treatment, and these institutions would have complete records of their cases."

He has, therefore, addressed his inquiries—which, by the way, are not in the slightest degree leading—(indeed, they do not even indicate any preconceived opinion on his part)—to the sources which, above all others, the average man would think were best able to furnish trustworthy data on the subject, but which the officials of the Agricultural Department, in the conduct of a recent similar investigation, refused even to consider, it being thought, by them, for some inscrutable reason, to use their own words, "that information from these sources would not be of a strictly representative character." And Dr. Boone brings his witnesses into court and makes them testify in their own verbatim language and over their own corporate and individual names.

A summarisation of the statistics and data contained in Dr. Boone's pamphlet shows that he received and publishes reports from 1,027 hospitals and sanitaria. Of these, 996 report that in all of their experience with the coal tar products there have been no instances of any untoward results, and that not a single case of habit formation from them has come under their observation. Injurious effects are reported by six hospitals only, all of which were due to overdose or other improper use of the remedies; seven institutions report cases, but state that they have no records, and therefore give no details; and one reports a case of insanity. The remaining seventeen out of the residuary thirty-one report cases of irregular pulse, weak heart action, cyanosis, and the like, under the administration of the drugs, none of which, however, were regarded as of enough importance to be noted in the report as serious, all of which were due to misuse of the drug, and all recovered. Not a single case of fatality is reported in the entire period covered by any one of the hospitals or sanitaria.

The scientific value of such an investigation and the trustworthiness of its evidence have only to be suggested in order to be immediately appreciated by any fair and unbiased mind. Here is an array of witnesses with no concealment of names or places, with no possible interest to subserve one way or the other, and hence with no thought of making a case for or against the products, each giving testimony from records that have been made with the careful accuracy which prevails in such institutions, all of which can be readily verified by any physician who cares to inspect those records, all set forth plainly and categorically, with no special pleading and with no conclusions or deductions, except those which the testimony itself forces upon the reader.

One can not fail to be impressed with the contrast offered by this investigation of Dr. Boone's to the methods employed and the showing made by the Bureau of Chemistry, under the direction of Dr. Wiley, in its recent investigation of the same subject

(referred to above), the results of which were published in its Bulletin No. 126, and whose specious and misleading conclusions Dr. Boone's inquiries were evidently designed to offset. A series of leading questions framed, as were those of Dr. Wiley, to elicit precisely the answers desired, and addressed to only 925 physicians, whose names are carefully withheld from the report and who, for all we know, may have been specially selected and prejudiced men, can hardly be regarded as the likeliest methods of obtaining impartial and trustworthy information upon this or any other subject.

The entire mass of evidence that filled this bureaucratic report was puerile, illogical and inconsistent; its testimony was incompetent; its facts were distorted, and its pleadings were so specious and prejudiced that they left no doubt in the mind of impartial readers of the predetermined purpose of the inquiry to condemn the products under the pretended investigation.

So strong was this prejudice, especially against acetanilid, that the most simply explicable data were twisted and distorted to serve its purpose, as for example, the explaining of the more extensive use of phenacetin, on the ground that it was the least harmful of the coal tar agents, when everyone with a grain of intelligence understands that, whatever excellence phenacetin may possess over acetanilid, its predominance in medical practice must be largely due to the fact that up to a very short time ago it was a proprietary, and hence was extensively and persistently advertised. In another place the bureau pointed out that the largest proportion of disasters occurred during the first eighteen months after the introduction of acetanilid, that in the next thirteen years the number of such disasters fell off, and that since 1904 there had been a notable increase in fatalities; and this it explains by the consideration that at first the dangers of the drug were not fully appreciated; that later, as it became better understood, it was used more carefully, and that of late years its use by the laity had given rise to increased fatalities. The true explanation, of course, is to be found in the fact that when acetanilid was a new remedy it was widely discussed and precisely reported on; and that as soon as the novelty wore off and its nature and action became thoroughly known, it naturally ceased to be the subject of frequent and detailed report and possibly was not used to quite the same extent as formerly.

And so the matter would have rested but for its agitation by the *Journal of the American Medical Association* and its lay allies—*Collier's Weekly* and the *Ladies' Home Journal*—during which the country was scoured for evidence, genuine or spurious, to bolster up their indictment against American medical specialties, and which represent precisely the five years or so in which the bureau pretended to find an increase in the number of fatalities.

. All of which is so transparent, and its instigation by the special interests of the medical ring so plain, that the only danger of the report lay in the color of authority given to it by the prestige of the United States Bureau. And all of which also is in marked contrast with the fair methods, the unbiased data and the straightforward presentation which characterises Dr. Boone's report.

The result of this orderly and competent investigation of Dr. Boone's is, as we have seen, precisely the reverse of the anomalous and incompetent inquiry conducted by the Department of Agriculture. Its net showing is, as any sane man would expect it to be, that the disasters and fatalities from acetanilid and the other preparations named have been no more and no less than those from other equally potent drugs; that, as a matter of fact, their untoward effects, as in the case of other powerful drugs, have been comparatively few; and that the beneficent effects of the coal tar products, including acetanilid, have been far in excess of their harmfulness.

It is immaterial to our criticism whether the subject under inquiry be acetanilid or any other product. What the medical and pharmaceutical professions are interested in is that investigations of drugs, by whomsoever undertaken, shall be fair and honest, which that of the Department of Agriculture can not be said to be, and which that of Dr. Boone's most assuredly is.

But without regard to the fairness and honesty of Dr. Wiley's investigation, we are unable to find any warrant in law for his proceedings in this matter. We do not understand by what or whose authority he has presumed to take it upon himself to advise the medical profession and the public in general as to what drugs they should or should not use. Nor are we aware of any statute which, however liberally construed, can be fairly said to give to the Agricultural Department the right to print and distribute at the public expense thousands and thousands of pamphlets in a propaganda against the coal tar products, *or for or against any other kind or class of drugs.*

But admitting the authority, we can conceive of no good reason why acetanilid, antipyrine and phenacetin should be singled out for special investigation and condemnation, as against many other drugs which are capable, if wrongly used, of producing at least equally harmful results. And in the absence of any such reason, and in view of the fact that Dr. Wiley is an enthusiastic member of the American Medical Association, is on one of its most important committees, and is outspokenly sympathetic in the fight which that association has waged against American medical specialties, we can not help feeling that he has allowed himself, innocently or otherwise, to be used as a tool to further the destructive schemes of the crafty medical clique at Chicago.

We believe that the investigation and report of Dr. Boone represents the real status of acetanilid and the other coal tar preparations. Indeed, we were satisfied that this was their status before any investigation was made at all; but we are sure that the manner and substance of the testimony presented by Dr. Boone is of such a character as to convince the fair and unprejudiced mind of the trustworthiness of its burden. Such an impartial and definite expression from the hospitals and sanitaria of the country ought to settle once and for all the vexed question of the danger and harmfulness of the coal tar products.

